# Prevalence of Different Head-Neck Positions in Horses Shown at Dressage Competitions and Their Relation to Conflict Behaviour and Performance Marks

**DOI:** 10.1371/journal.pone.0103140

**Published:** 2014-08-04

**Authors:** Kathrin Kienapfel, Yvonne Link, Uta König v. Borstel

**Affiliations:** 1 Department of Animal Ecology, Evolution and Biodiversity, Ruhr Universität Bochum, Bochum, Germany; 2 Institute of Animal Breeding and Husbandry, Christian-Albrechts University of Kiel, Kiel, Germany; 3 Department of Animal Breeding and Genetics, Georg-August-Universität Göttingen, Göttingen, Germany; University of Portsmouth, United Kingdom

## Abstract

Much controversy exists among riders, and in particular among those practicing dressage, regarding what can be considered an “appropriate” Head-Neck-Position (HNP). The objective was to assess the prevalence of different HNPs in the field, the behavioural reactions of horses during warm-up and competition rides in relation to HNP and the relation between HNP and marks achieved in the competition. Horses (n = 171) were selected during dressage competitions according to their HNP (3 categories based on the degree of flexion), and their behaviour was recorded during 3 minutes each of riding in the warm-up area and in the competition. Scans were carried out on an additional 355 horses every 15 minutes to determine the proportion of each HNP in the warm-up area. Sixty-nine percent of the 355 horses were ridden with their nasal planes behind the vertical in the warm-up area, 19% were ridden at or behind the vertical and only 12% were ridden with their nasal plane in front of the vertical. Horses carrying their nasal plane behind the vertical exhibited significantly (P<0.0001) more conflict behaviours than horses with their nose held in front of the vertical. Horses were commonly presented with a less flexed HNP during competition compared to warm-up (P<0.05). A HNP behind the vertical was penalised with lower marks in the lower (P = 0.0434) but not in the higher (P = 0.9629) competition levels. Horses in higher classes showed more (P = 0.0015) conflict behaviour than those in lower classes. In conclusion, dressage horses are commonly ridden during warm-up for competitions with their nasal plane behind the vertical, and this posture seems to cause significantly more conflict behaviour than HNPs in front of the vertical.

## Introduction

Riding styles, and in particular horses' postures during riding commonly are subject to welfare debates. While there is little doubt that riding horses with extremely elevated heads entails welfare issues [1], in the past years there has been much discussion regarding the so-called “classical” and the “modern” riding style. With regard to the HNP the “modern” riding technique is also referred to as rollkur, hyperflexion or LDR (Low, Deep and Round). It has been practiced at least as early as in the 17^th^ century [2]. The “classical” riding style was summarized and codified in the German military regulations from 1912 [3]. These regulations include the rule that the horses' nasal plane should be slightly in front of the vertical.

Today both riding techniques are used. However, especially the “modern” technique is a controversial issue and there is an urgent need for an objective assessment of its implications. Pfeil-Rotermund & Zeeb emphasized in their study the expression and conflict behaviour of show jumping horses [4]. They showed that recording the influence of the rider on the horse with ethological methods is possible. Caanitz studied the horses' expressive behaviour at the beginning of their training as a result of the interaction between horse and rider [5]. She found, that horses which hold the nasal plane behind the vertical express more defensive behaviour than those carrying their nasal planes in front of the vertical. These results were confirmed by other studies [6], [7], [8]. Horses ridden in rollkur or LDR showed more signs of discomfort, such as tail-swishing, abnormal oral behaviour and ears fixed back, than horses ridden in “normal” poll flexion with the nasal plane mostly in front of the vertical, a posture also termed as “competition frame” [6], [7]. Horses not only express higher levels of discomfort when ridden in rollkur-posture, they also avoided rollkur, if given the opportunity [6]. One study suggested that a reason for increased discomfort during rollkur may be the restricted vision [9]. The latter has also been held responsible for more pronounced fear reactions due to higher levels of arousal or anxiety in horses ridden in rollkur-positions compared with normal poll flexion [6].

The aim of the present study was to quantify behaviour patterns in horses competing at dressage competitions, and to assess potential differences between HNPs and competition levels. Of particular interest was the influence of HNP on the achieved mark in the competition. In view of the study of Pfeil-Rotermund & Zeeb, we expected a difference in conflict behaviours between levels of dressage [4].

## Material and Methods

### Ethics statement

This type of non-invasive, behavioural research is approved under the German animal protection act and does not require a study-specific permission. The authors did not manage or handle the horses in any way. Riders of the horses were not informed about the study as this information might have biased results. Researchers did not interfere in any way with riding styles, horses or riders, and all riding corresponded to the routine competition procedures. No additional activity was undertaken for the purpose of this study.

### Pilot study

In order to assess the potential influence of bias in the observer from the main study, a pilot study was conducted on 29 horses from one dressage competition not included in the main study, using two different observers: one of these observers was familiar with horses, while the other was entirely unfamiliar with horses. However, as students of biology both were familiar with taking behavioural observations. The two observers were recruited in class at university, they provided written consent for the use of their observations in this study, and they thus knew that they were participating in research. Methods for behavioural observations were the same as described below, but observations were made only in the warm-up area. Also in contrast to the main study, but in accordance with an earlier study [7] HNPs were grouped only into two categories: the horse carries the nasal plane in front of the vertical or the horse carries the nasal plane behind the vertical. Due to data loss, observations from this pilot study could not be traced back to individual observers. Therefore, an additional part of pilot study was conducted. It included as observer the person from the main experiment as well as an additional observer unfamiliar with horses, who likewise provided written consent and knew that he was participating in research. Both persons observed the videos of 5 different horses during dressage competitions unrelated to those involved in the main experiment, but selected to include the full range of HNPs observed in the main study. Both observers used the same HNP classifications and ethogram as described for the main experiment. Inter-observer reliability for the total number of conflict behaviours as well as for the individual conflict behaviours tail-swishing, abnormal oral behaviour and change in gait was calculated based on variance components of a generalized linear mixed model analysis [7] using observer as a fixed factor and horse as a random factor. Inter-observer agreement for the categorical variable HNP, as well as for the behaviours with rare occurrence (all other behavioural variables) were assessed as percentage of agreement between the two observers.

### Main study

During a total of 180 rides, dressage horses (n = 171) were observed at eleven local, national and international dressage events. Due to the convenience sampling, nine horses were observed twice during two independent rides. Horses varied in sex, age and breed, but were mostly warmbloods. They took part in different types of dressage classes at German levels A, L, M and S (approximately corresponding to novice, elementary, medium and advanced levels e.g. in the New Zealand classification system). Levels were combined into two groups with lower (A–L) and higher (M–S) dressage performance level. Observed rides were selected for the study based on horses' HNP during warm-up, with 30 horses each per HNP-class as described below within each of the two performance level groups (A–L and M–S). Horses were subjectively categorised into one of three possible HNPs:

1. Nasal plane mostly in front of the vertical (“IV”) (maximum flexion within a ride such that the nasal plane is at the vertical)
2. Nasal plane slightly behind the vertical up to ten degrees (“AV”) (minimum flexion within a ride such that the nasal plane is at the vertical)
3. Nasal plane more than ten degrees behind the vertical (“BV”)

Compared to the pilot study, the HNPs behind the vertical were further distinguished into two categories, to allow for a more accurate differentiation between different degrees of flexion.

The observation of the conflict behaviour and the HNPs was carried out during the phases of active riding, when the reins were short and the horse did not stand relaxed. In order to be assigned to one of the above categories, horses had to spend an approximate minimum of 95 percent of the three-minute interval in the respective HNP. Horses that were ridden less than three minutes continuously or with an unsteady, variable HNP, that did not fit clearly into one of the categories above, were excluded. The horses were observed live for 3 minutes in the warm-up area and again for the first 3 minutes of the test (starting immediately after the start signal). According to a previously validated method [8], during both observation periods, the frequency of individual behaviour patterns (table 1) potentially indicative of discomfort or conflict was recorded, and in addition the sum of all behaviour patterns calculated. From here on in the current text, these responses will be referred to as “conflict behaviour”.

**Table 1:** Ethogram of the observed behaviour acts (adapted from [6], [8]).

Behaviours	Description
Tail-swishing	Fast movements of the tail
Ears fixed back	Ears are >5 sec fixed back
Head tossing	Fast up and down movement of the head
Mouth wide opened	The mouth is >3 sec. as wide open as the noseband permits
Showing teeth	Lips pulled back showing teeth for >3 sec.
Abnormal oral behaviour	Chewing with an open mouth, showing tongue
Nose tilting	The horse tilts its nose to one side
Going-against-reins	The horse pulls against the reins and breaks the line between elbow of the rider and rings of the bit
Change in gait	The horse changes the gait
Crabbing	Hind legs of the horse do not follow the track of the front legs
Attempts to buck	The horse shifts its weight on the front legs and moves the hind legs upwards

In a second part of the experiment, for a total of 26.25 hours, scan sampling every 15 minutes (105 times) was conducted on eight days of three dressage events. All horses (N = 355 in total) present at that time in the warm-up area were classified according to their HNP. These scans were made only during warm-up before the tests at the levels L, M and S.

Marks at the levels A and L are given on a scale from 1 to 10, while most of the marks given at levels M and S are reported in percent. Therefore, marks reported in percent were transformed (i.e. divided by ten) to obtain marks that are displayed on a comparable scale, and thus to allow for a combined analysis. All statistical analyses were conducted with SAS 9.2 (SAS Institute Inc., Cary, North Carolina, USA, 2009). With the exception of these marks (Kolmogorov-Smirnov: P = 0.1110), data were not normally distributed (P<0.05). Therefore, marks were analysed with a mixed model accounting for repeated observations per horse. Frequencies of conflict behaviour were analysed with a generalized linear mixed model, assuming either an underlying Poisson (total number of conflict behaviour and tail-swishing), binary (head-tossing and bucking) or overdispersed Poisson (all other variables) distribution. Model fit was verified based on scaled Pearson statistics. In all cases, the effect of the fixed factors HNP (in front, at or behind the vertical) as well as the situation (warm-up or test; except for the analysis of marks), the level group (A/L and M/S) and their interactions were tested. The tukey-adjustment was used to adjust for multiple comparisons. With the analysis of the marks, in addition the numbers of behaviours observed during warm-up (total number only) and during the test (total number and individual conflict behaviour) were each considered in a separate analysis as a covariate. Fisher's exact test was used to assess separately by competition level group the significance of deviations from expected percentages of HNPs during test situation in relation to HNP during warm-up.

## Results

### Pilot study

The independent observers classified 43.4% of the 29 observed horses to be ridden with an HNP in front of the vertical and 58.6% with the nasal plane behind the vertical. According to their observations, the horses ridden with an HNP in front of the vertical showed on average 10.6 behavioural acts as listed in table 1 per three minutes in the warm-up area, while the horses ridden with an HNP behind the vertical showed on average 23.3 behavioural acts. Inter-observer reliability for the total number of conflict behaviours was 0.99±0.01. For tail-swishing, abnormal oral behaviour and gait change it was 0.99±0.01, 0.90±0.10 and 0.43±0.46, respectively. Inter-observer agreement for head-tossing was 80%, and for the remaining conflict behaviours as well as HNP it was 100%.

### Main study

#### Marks

**Figure 1:**
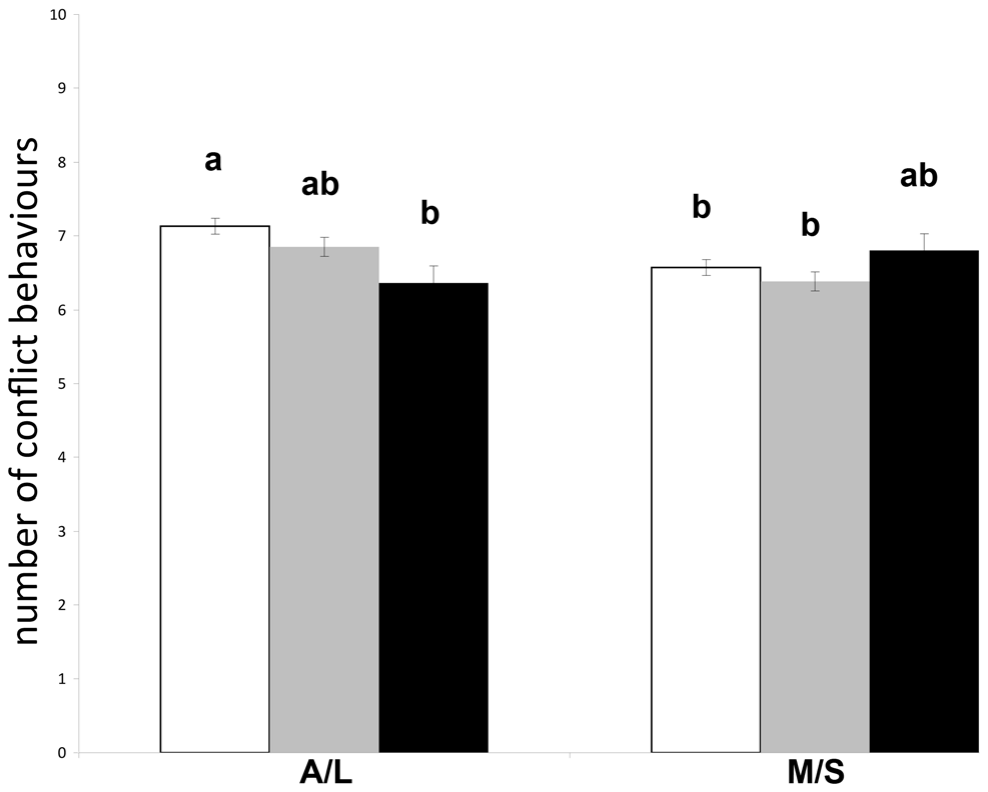
Marks: least square means (± SE) of dressage marks by level group (lower levels A and L versus higher levels M and S) and by head-neck position (white  =  nasal plane in front of the vertical, grey  =  nasal plane at the vertical, black  =  nasal plane behind the vertical). Different letters a,b indicate statistically significant differences at P<0.05.

The interaction between group level and HNP was significant (P = 0.0002) such that riders using a more flexed HNP were awarded lower marks in the lower levels (A/L), but not in the higher (M/S) levels (Figure 1). The total number of conflict behaviours shown during warm-up (P = 0.2438) or during the test (P = 0.4236) did not influence the marks. Of the individual conflict behaviours shown during the test, only the frequency of “going against the reins” tended to influence marks (−0.16±0.08 marks per additional occurrence of the behaviour pattern; P = 0.0504), and horses that bucked (5.8±0.52) tended to receive lower scores than horses that did not buck (6.7±0.09) during the test (P = 0.0675).

#### Behaviour

**Figure 2:**
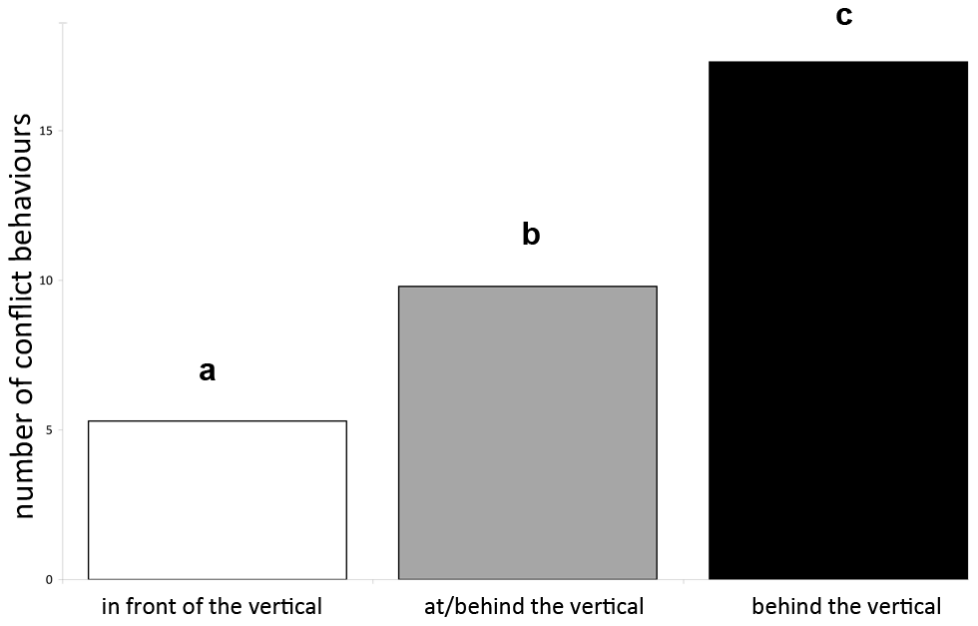
Behaviour: mean (re-transformed least square means) total frequency of conflict behaviour per 3-min. ride depending on the head-neck position (white  =  nasal plane in front of the vertical, grey  =  nasal plane at the vertical, black  =  nasal plane behind the vertical). Different letters a,b,c indicate significant differences at P<0.0001.

The total number of conflict behaviours was significantly influenced by the HNP (P<0.0001) and the level (P = 0.0015), but not by the situation (warm-up or test; P = 0.5907). Horses ridden “IV” showed significantly fewer conflict behaviours, compared to those in the categories “AV” or “BV” (P<0.05) (Figure 2). Horses in the higher levels (re-transformed least-square mean frequency of conflict behaviours per three minutes of riding: 12.5) showed significantly (P = 0.0014) more conflict behaviours compared to horses in the lower levels (9.3 behaviours). The interaction between HNP and level was not significant (P = 0.2776), indicating that the effect of the different HNPs was similar at both the lower and higher levels.

**Figure 3:**
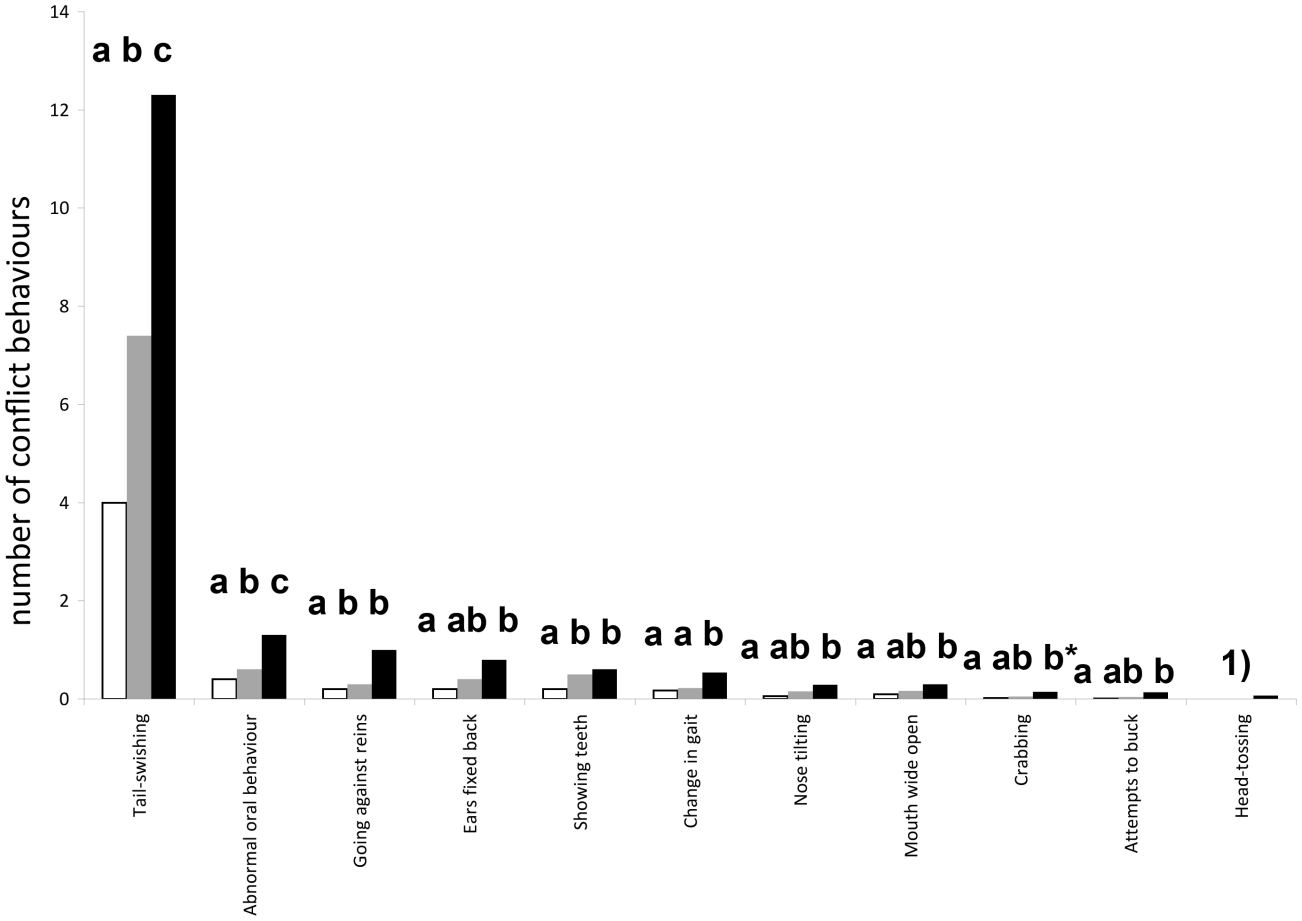
Individual conflict behaviour: mean (re-transformed least square means) frequency of individual conflict behaviour per 3-min. ride depending on the head-neck position with the nasal planes in the respective categories. Different letters a,b,c indicate statistically significant differences at P<0.05 (*P<0.1). 1) Effect not estimable due occurrence in just one category (HNP behind the vertical).

When considering the individual behaviour acts, the same trend of higher frequencies of conflict behaviour in more flexed HNPs can be observed (Figure 3). Only for mouth-opening, no significant (P>0.1) overall effect of HNP on frequencies of behavioural acts could be found. Head-tossing could not be statistically compared, because it was only observed in the category “BV”. In addition to HNP, the situation influenced a number of individual behaviour acts. Loss of rhythm (P = 0.0570) and unusual oral behaviour (P = 0.0240) occurred more frequently during the test compared to the warm-up. Head-tilting (P = 0.0243) and ears pointed backwards (P<0.0001) occurred more frequently during warm-up compared to the test. Going against the reins was shown more often (P<0.0001) in the lower level (A/L: re-transformed least square mean frequency: 0.7 times/ride) compared to the higher level (M/S: 0.3 times/ride) horses, while tail-swishing was shown approximately twice as often in the higher-level horses (10.1) compared to the lower level horses (5.8; P<0.0001).

#### Change in head-neck position from warm-up to competition

**Figure 4:**
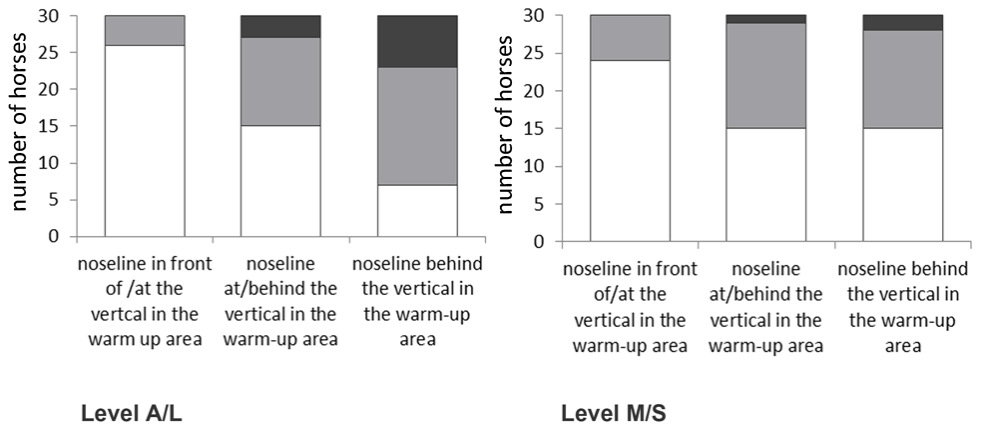
Distribution of head-neck-positions: distribution (number of horses) of head-neck positions (HNP) in competition by HNP in the warm-up area. HNP in competition: white  =  nasal plane in front of the vertical, grey  =  nasal plane at the vertical, black  =  nasal plane behind the vertical. At both group levels, HNP distributions deviated significantly from chance, with more riders than expected presenting their horses in competition with an HNP in front of the vertical (A/L: P<0.0001, M/S: P = 0.0479).

Both for lower (Fisher's exact test: P<0.0001) and higher (Fisher's exact test: P = 0.0479) level horses, the distribution of HNPs during competition deviated significantly from that expected by chance (i.e. an equal distribution). Regardless of the HNP used during warm-up, riders rarely (A/L: 11%; M/S: 3% of the observed rides) presented their horses during competition with a HNP BV (Figure 4). Instead, in more than half of the rides (A/L: 53%; M/S: 54% of the observed rides) horses were presented during competition with an HNP in front of the vertical. In particular, those riders who warmed-up their horses with an HNP in front of the vertical, rarely made changes to this HNP when entering the competition (in 87% (A/L) and 80% (M/S) of these rides the HNP remained unchanged in front of the vertical).

#### Scans, frequency of hyperflexion

**Figure 5:**
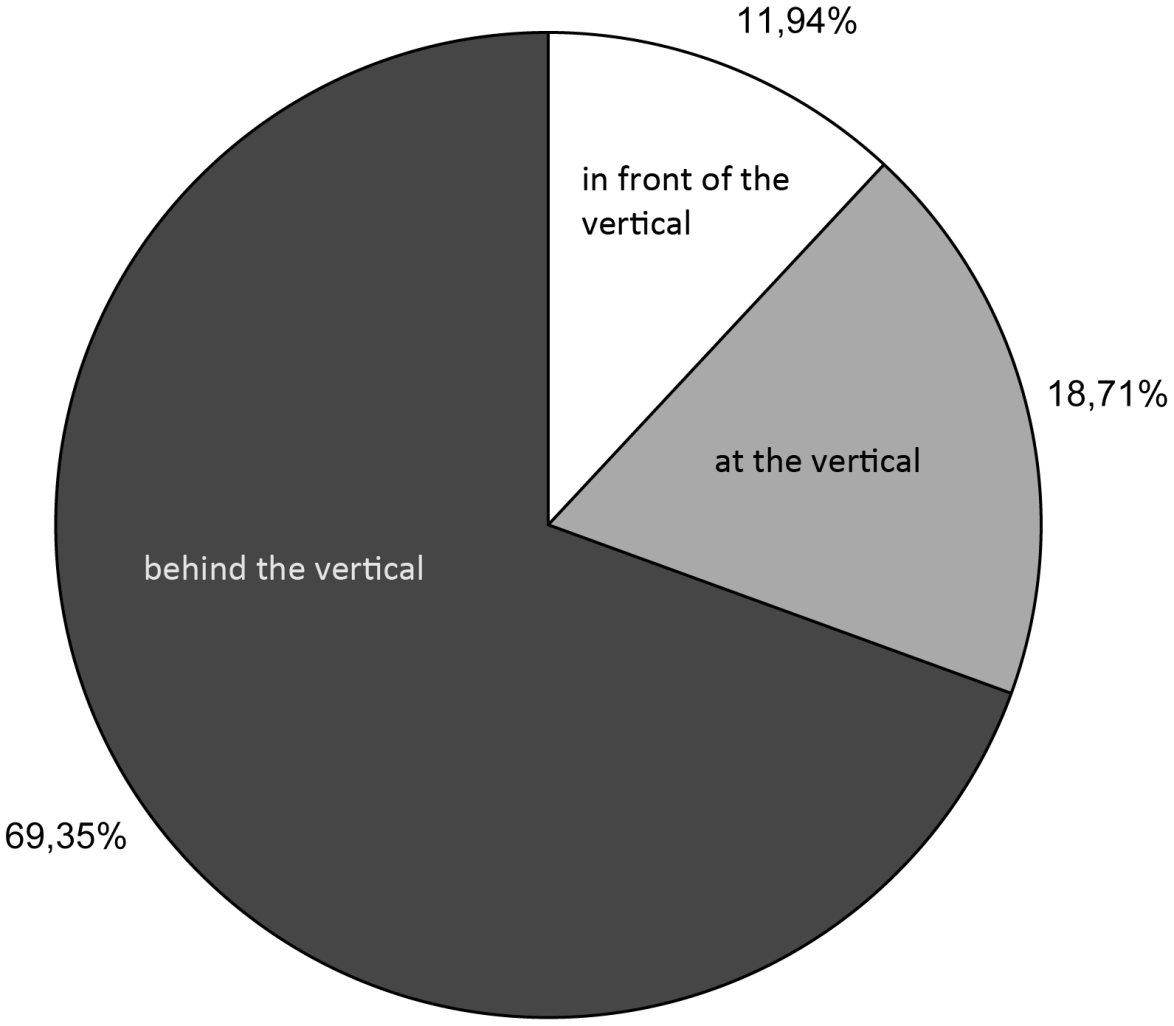
Behavioural Scans: proportions of horses ridden with the different Head-Neck-Positions with the nasal planes in the respective categories at the warm-up area.

Of the 355 horses observed during warm-up, 11.9% were ridden with their nasal planes in front of the vertical. Only 18.7% of the horses were ridden with an HNP at the vertical, and 69.4% of the horses were ridden with an HNP behind the vertical (Figure 5).

## Discussion

Regardless of the competition level, horses exhibited more conflict behaviours the more the head was kept behind the vertical. Other studies obtained similar results, supporting the assumption that a strongly flexed HNP is perceived by horses as uncomfortable [6], [8], [9], [11]. Contrary to claims by some practitioners and/or scientists (e.g [30]), the lack of an interaction between HNP and level indicates that a strongly flexed HNP has similar effects on the horse, regardless of whether it is achieved by a more or a less proficient rider.

Tail-swishing may be due to different reasons. In a completely “natural” environment it is a defense reaction to flies. But an unusual infestation of flies as an explanation for tail-swishing in certain horses can be ruled out, because all horses were exposed to the same conditions. Tail-swishing can also be a reaction to the use of spurs and a whip [12]. Therefore its increased frequency in horses ridden with an HNP more strongly behind the vertical can be an indication that these horses are exposed more often to the forceful actions of legs, spurs and whips, as also observed in the study by von Borstel *et al*. [6]. Taken together, these findings indicate that horses with a more strongly flexed HNP may be ridden in a more aggressive fashion [8]. In addition tail-swishing is linked to situations of fear and pain [12]. Another study showed that a very low, deep, round HNP induces a state of heightened arousal or anxiety, and in that study tail-swishing also occurred more frequently during hyperflexion [6]. According to McGreevy tail-swishing seems to be a typical response to a conflict [10]. Generally, shortening the reins (as required to achieve a hyperflexed HNP) leads to higher rein tensions [11]. Since increases in rein tension are used as a stop signal the simultaneous increase in the use of whip and spurs, which are mainly used as “go” signals, are likely to induce such a state of conflict in the horse [13]. Kienapfel found “mouth wide open” to be the second most frequent behaviour [8]. This result could not be confirmed in the present study. Potentially the routine use of tight nosebands prevented the horses from showing this behavioural act more frequently [14], [15]. In another study, 47% of jumping horses had the nosebands fastened so tight, that no finger fitted between the leather and the nasal bone [16], i.e. considerably tighter than the allowed minimum of two fingers. For dressage horses, unfortunately no comparable data are available yet.

The increase of gait changes and loss of rhythm and regularity of gait could likewise be an indicator for dissonant riding aids. Another explanation may be found in the principles of locomotor mechanics of the horses. A flexion of the horse's neck modifies the gaits [17], [18], [19]. This may be because of or in addition to the higher physiological “workload” in hyperflexion [20]. Whether the workload is so high, that changes in gait and loss of rhythm and regularity are correlated, remains to be examined.

Becker-Birck et al. found no differences in the behaviour of lunged horses without riders in different HNPs, where the HNPs were achieved with draw reins [21]. The same pattern occurred in the study of Kienapfel [22]. Horses, which were lunged in different HNPs showed subjectively few signs of discomfort in the hyperflexed position. In our study and other studies without draw reins, the opposite effect could be seen [6], [8], [9]. Becker-Birch's observation is possibly an effect of “learned helplessness” [21]; all horses were used to draw reins at lunging and had possibly learned that there is no way to avoid the adjusted HNP.

Many riders prepared their horses with the nasal plane “BV”, but the prevalence was significantly lower during competition, indicating that the majority of riders might be aware of what is the correct HNP according to the guidelines. Apparently the riders pay more attention to HNP of their horses when under the eyes of the judges. Potentially, riders also perceive their horse to be more relaxed or willing during competition, when they release their horse from a more flexed HNP during warm-up to a less flexed HNP just before the start of the test. In particular when horses were ridden with the head “BV” in the warm-up area, riding style changed markedly in competition. Only 23.3% of the horses at levels A and L were ridden during competition in the same way as in the warm-up area. This group of less advanced riders might not have the feeling for the right head position, or their teachers do not correct this way of riding. At levels M and S, 6.7% of the riders presented their horses in the competition with the horse's head “BV”. Riders at these levels supposedly know that riding in a HNP behind the vertical should, according to the FEI-guidelines (“The head should remain in a steady position, as a rule slightly in front of the vertical, with a supple poll as the highest point of the neck, and no resistance should be offered to the Athlete.” [22]) lead to lower marks for their performance, which may be the reason for them to change the HNP in the competition. Interestingly, according to our results judges appeared to penalize deviations in the HNP from the ideal with lower marks in the lower, but not in the higher classes according to the guidelines. Although the low proportion of strongly flexed HNPs during competition in the higher levels may partially bias results, these findings raise the question whether judges potentially pay less attention to such aspects in the higher classes. Considering that riders in the higher levels serve as examples, if not idols, for riders of the lower classes, these findings are rather alarming. It seems difficult to justify, why rules are applied differently to riders of higher and lower levels.

### Marks

Riding style in the warm-up area did not influence marks obtained in competition. Therefore, a warm-up with a HNP BV either does not lead to lasting tensions in the horses that would compromise performance in competition, even if the riders then changed to a less restrictive HNP, or judges do not penalize this potential tension. Plewa explained that a short and tight neck resulting in a lack of relaxation, change in gaits and the horse's behaviour can influence the mark [23]. However, this influence on marks could not be detected in the present study. The general lack of influence of the frequency of conflict behaviour on marks is likewise an alarming finding. Either, judges ignored these behavioural signs of discomfort or horses' discomfort is associated antagonistically with other parameters of performance.

In competitions at level S the marks for “suppleness” (how the horse reacts to the rider aids) and “contact” (connection to the horses' mouth) are the most variable marks in the final grading [24]. Suppleness is thought to be a sign of a content horse and therefore should be connected with low levels of discomfort, so assessing these relationships based on detailed marks would be interesting for future studies.

Also, overall marks improve with a longer preparation time [25]. Specifically which aspects are affected by preparation time, and if there is a relationship between the specific marks for suppleness and preparation time has yet to be studied.

### Levels

According to earlier studies, in show-jumping [4], as well as in dressage and western [26] horses in lower classes show more conflict behaviour than horses in higher classes. In contrast, in the present study as well as in the dressage horses observed by Pfeil-Rotermund & Zeeb [4], the frequency of conflict behaviour increased with higher levels. The assumption that incorrect riding aids [4] and thereby the less correct application of learning theory [26] in lower level riders induced the defensive behaviour, does not seem to be applicable for the competition context as studied in the present study as well as in the study by Pfeil-Rothermund and Zeeb [4]. Rather, it seemed to be that during competitions, the riders in higher classes rode much more aggressively, thus outweighing any beneficial effects of an improved seat and a more controlled application of riding aids. Measures of spur and whip use could be included in future research to investigate these aspects further.

### Scans

Although in an earlier study, Kienapfel used only two different HNPs to group horses in warm-up areas, her results are comparable and in agreement with the present study [8]. She observed that 92.8% of the horses belonged in the group “BV”. Comparable to this group are all horses with the nasal plane “BV” and “AV” of the present study, which comprised 88.06% of all horses in the warm-up areas.

These results are backed by studies showing that photographs of horses advertised for sale in magazines or trading websites, were presented in 68% [27] and 70% [28] of the cases, respectively, with their nasal planes behind the vertical. Presumably, these horses were presented at their best according to the vendors' point of view [28].

In summary, approximately 90% of all dressage horses are ridden in the warm-up area with the nasal plane BV, i.e. a style which does not conform with the rules of the FEI (FEI-Rules, Chapter 1, Article 401, 5).

### Method

As there was only one observer in the present study and because the observer was not blinded to the HNP group, observer bias might have been present, posing some constraints to the present study. On the other hand, results from the pilot study suggest that other observers, including two entirely unfamiliar with horses, obtain similar results. With the exception of gait change, which may be difficult to recognize for people unfamiliar with horses, inter-observer reliability and inter-observer agreement was very high, and indeed generally higher than inter-observer reliabilities from other behavioural research in horses [29], [30], [31]. Therefore, these results suggest that any observer bias, if present, was of limited extent. However, in future studies that include observations of the conflict behaviour “gait change”, particular attention should be paid to this behaviour, which may require a more thorough definition to allow also laypersons to identify it correctly.

## Conclusions

Results of the present study indicate that horses are frequently ridden at dressage competitions with an HNP behind the vertical, although more commonly so during warm-up compared to the competition itself. Such a flexed HNP is penalized with lower marks in the lower, but not in the higher competition levels. Furthermore, the current results indicate that horses show conflict behaviour more frequently when ridden with an HNP behind the vertical rather than at or in front of the vertical. Since this effect was observed regardless of the competition level, it is suggested that the flexed posture itself rather than the rider's skills or the training level of the horse is the main factor inducing conflict behaviour.
